# *ZNF25* as an immunotherapy target: pan-cancer biomarker potential and mechanistic exploration in glioma

**DOI:** 10.3389/fonc.2026.1631383

**Published:** 2026-06-02

**Authors:** Xiaohong Yi, Xianwen Zhang, Lijun Huang, Yuhui Chen, Maochun You, Hongyi Jia, Lefeng Hou, Chao Hu, Kun Huang, Yumei Wang

**Affiliations:** 1 School of Basic Medical Sciences, Chengdu University of Traditional Chinese Medicine (CDUTCM), Chengdu, China; 2 Chinese Medicine Germplasm Resources Innovation and Effective Uses Key Laboratory of Sichuan Province, Chengdu, China; 3 Department of Neurosurgery, The Second Affiliated Hospital of Guangzhou Medical University, Guangzhou, Medical University, Guangzhou, China; 4 Department of General Surgery, Mianyang Hospital of Traditional Chinese Medicine, Mianyang, Sichuan, China

**Keywords:** *ZNF25*, pan-cancer analysis, glioma, PI3K-AKT pathway, RNA-seq

## Abstract

**Background:**

*ZNF25*, a KRAB domain-containing zinc finger transcription factor, has been identified as a potential biomarker in ovarian cancer. However, its pan-cancer significance and the underlying molecular mechanisms in other malignancies remain largely unclear.

**Methods:**

We performed a systematic pan-cancer analysis of *ZNF25* using TCGA and GTEx databases, integrated with platforms including cBioPortal, TISIDB, TIMER, and Sangerbox. Associations between *ZNF25* expression and clinical outcomes, genomic alterations, immune cell infiltration, and DNA methylation were systematically evaluated. Based on these findings, functional validation was performed in glioma cell lines (U87-MG and A172) via siRNA-mediated knockdown of *ZNF25*, followed by cell viability assays, wound healing assays, and Western blot assays. Additionally, RNA-seq was conducted in *ZNF25*- knockdown U87-MG cells to identify differential expression genes and enriched pathways.

**Results:**

*ZNF25* exhibited significant associations with diagnostic potential, poor prognosis, and immune infiltration across multiple cancer types, with the most pronounced effects observed in glioma. Functional assays demonstrated that *ZNF25* knockdown suppressed the proliferation and migration of glioma cells. Transcriptomic and biochemical analysis indicated that *ZNF25* promotes glioma progression, at least in part, through the positive modulation of the PI3K-AKT pathway.

**Conclusions:**

Collectively, our findings identify *ZNF25* as a potential diagnostic and prognostic biomarker across various cancers and suggest its critical role in glioma progression. This study provides new insights into the functional role of *ZNF25* and highlights its potential as a therapeutic target, particularly in glioma.

## 1 Introduction

Cancer is the second leading cause of death worldwide, with approximately 10 million deaths in 2022 (1). Currently, cancer can be treated with a multifaceted approach encompassing surgical interventions, radiotherapy, chemotherapy, targeted therapy, and immunotherapy. Nevertheless, clinical outcomes for many individuals remain unsatisfactory, mainly due to delayed diagnosis, high recurrence risk, severe treatment-related adverse effects, and drug resistance (2, 3). Immunotherapy has brought about a substantial transformation in the field of cancer treatment, notably through the remarkable success of immune checkpoint inhibitors (ICIs) and Chimeric Antigen Receptor T-Cell Therapy (CAR-T cell) therapy clinical applications (4). Nonetheless, patients receiving identical regimens often exhibit highly variable response rates, largely driven by intertumoral heterogeneity and differences in the tumor immune microenvironment (TIME) (5). Consequently, identifying novel biomarkers for precise diagnosis and prognosis is urgently needed. The rapid advancement of immunotherapy further underscores the importance of discovering and validating immune-related biomarkers.

Transcription factors play a crucial role in the development of various cancers, serving as key proteins that regulate gene expression. Zinc finger proteins with KRAB domains (KRAB-ZNF) constitute the largest transcriptional regulatory factor family in human, with most members clustered throughout the genome (6). Their structural hallmark includes an N-terminal KRAB domain and multiple C2H2-type zinc finger domains at the C-terminus (7). They not only participate in normal biological processes, including transcriptional regulation, organogenesis, and cell differentiation, but also play a significant role in tumors (6, 8). However, further research is needed to elucidate their specific mechanisms and roles in different types of cancer.

*ZNF25*, also known as *KOX19*, and *ZFP9*, is a member of the C2H2 zinc finger protein family. Located on human chromosome 10, *ZNF25* encodes a protein with multiple zinc finger motifs, exhibiting widespread expression across various tissue (9). Its primary mechanism is to act as a transcription factor, capable of binding to specific DNA sequences and negatively regulating transcription by RNA polymerase II. Research has shown that *ZNF25* participates in neural development and differentiation, osteoblast differentiation, and transcriptional deregulation in the retrosplenial cortex caused by anterior thalamic lesions (10, 11). Additionally, studies suggest that dysregulation of *ZNF25* may be associated with ovarian cancer and esophagus cancer (12, 13).

However, reports on *ZNF25* in other cancer types remain scarce, and its detailed molecular mechanisms are poorly understood. Despite its potential implication in cancer, the multifaceted role of *ZNF25*, particularly its influence on tumor immunity and clinical outcomes, remains poorly understood across different cancers.

This study conducted an extensive pan-cancer analysis of *ZNF25*, and elucidated the significant influence of *ZNF25* on tumor diagnosis, prognosis, and TIME, etc. We found *ZNF25* knockdown can inhibit the proliferation and migration of U87-MG and A172 cells. Remarkably, we also performed RNA sequencing (RNA-seq) in *ZNF25*-knockdown U87-MG cells, differentially expressed genes (DEGs) were greatly enriched in PI3K-AKT signaling pathway. *ZNF25* knockdown reduced p-AKT protein levels both in A172 and U87-MG cells. These findings underscore *ZNF25* as a promising cancer biomarker, and candidate target for cancer therapy, with special relevance to glioma.

## 2 Materials and methods

### 2.1 Data collection

Gene expression data, clinical data, and mutation data of *ZNF25* across 41 types of cancer and their matched normal tissues were downloaded from the UCSC Xena database (https://xena.ucsc.edu/) (14), GTEx database (https://www.genome.gov/Funded-Programs-Projects/genotype-Tissue-Expression-Project) (15). The full names and abbreviations of the 41 cancer types are listed in **Table 1**. In this study, “glioma” refers to diffuse gliomas of the brain (WHO grades II–IV). Glioma data were sourced from The Cancer Genome Atlas (TCGA) project under the identifier “GBMLGG”, which consolidates two major subtypes: lower-grade gliomas (LGG, grades II–III) and glioblastoma multiforme (GBM, grade IV), excluding other glioma entities such as pilocytic astrocytoma and spinal cord gliomas. The terms GBM, LGG, and GBMLGG are used consistently in accordance with the TCGA classification standards.

**Table 1:** Full names and abbreviations of the 41 cancer types in TCGA.

Abbreviation	Cancer types
ACC	Adrenocortical carcinoma
BLCA	Bladder Urothelial Carcinoma
BRCA	Breast invasive carcinoma
CESC	Cervical squamous cell carcinoma and endocervical adenocarcinoma
CHOL	Cholangiocarcinoma
COAD	Colon adenocarcinoma
COADREAD	Colon adenocarcinoma/Rectum adenocarcinoma
DLBC	Lymphoid Neoplasm Diffuse Large B-cell Lymphoma
ESCA	Esophageal carcinoma
GBM	Glioblastoma multiforme
GBMLGG	Glioblastoma and Lower Grade Glioma
HNSC	Head and Neck squamous cell carcinoma
KICH	Kidney Chromophobe
KIPAN	Pan-kidney cohort (KICH+KIRC+KIRP)
KIRC	Kidney renal clear cell carcinoma
KIRP	Kidney renal papillary cell carcinoma
LAML	Acute Myeloid Leukemia
LGG	Brain Lower Grade Glioma
LIHC	Liver hepatocellular carcinoma
LUAD	Lung adenocarcinoma
LUSC	Lung squamous cell carcinoma
MESO	Mesothelioma
OV	Ovarian serous cystadenocarcinoma
PAAD	Pancreatic adenocarcinoma
PCPG	Pheochromocytoma and Paraganglioma
PRAD	Prostate adenocarcinoma
READ	Rectum adenocarcinoma
SARC	Sarcoma
STAD	Stomach adenocarcinoma
SKCM	Skin Cutaneous Melanoma
STES	Stomach and Esophageal carcinoma
TGCT	Testicular Germ Cell Tumors
THCA	Thyroid carcinoma
THYM	Thymoma
UCEC	Uterine Corpus Endometrial Carcinoma
UCS	Uterine Carcinosarcoma
UVM	Uveal Melanoma
OS	Osteosarcoma
ALL	Acute Lymphoblastic Leukemia
NB	Neuroblastoma
WT	High-Risk Wilms Tumor

### 2.2 Differentially expressed and survival analysis of *ZNF25* across cancers

The expression data of the *ZNF25* gene was extracted and log_2_(x+0.001) transformed. Cancers types with fewer than 3 samples were excluded from analysis. The mRNA expression of *ZNF25* in tumor tissues, adjacent normal tissues, and different pathological grades were analyzed using the Sangerbox platform (http://sangerbox.com/home.html) (16). Differentially expressed results were assessed using the Wilcoxon rank-sum test (unpaired samples) and Wilcoxon signed-rank test (paired samples), with adjusted *P* < 0.05 considered statistically significant.

The correlation of *ZNF25* expression and clinical outcomes, including Overall Survival (OS), Disease-Specific Survival (DSS), and Progression-Free Interval (PFI) was evaluated across cancers. Forest plots were generated using the SRplot web server (http://www.bioinformatics.com.cn/SRplot) (17), and P-values were adjusted using p.adjust function in R (version R 4.5.0).

TIMER2.0 (http://timer.cistrome.org/) is a comprehensive resource for systematic analysis of immune infiltrates across diverse cancer types (18). The “Gene Outcome” module of Exploration in TIMER2.0 was used to evaluate the prognosis significance of *ZNF25* expression via Cox proportional hazard regression. Survival differences between *ZNF25* high- and low-expression groups (stratified by median expression) were analyzed using the log-rank test to generate Kaplan–Meier curves.

### 2.3 Association analysis between *ZNF25* and tumor genomic features

Tumor mutational burden (TMB) (19), microsatellite instability (MSI) (20), and neoantigen load (NEO) (21) are associated with tumor immunogenicity and predict favorable response to immune checkpoint inhibitors. In contrast, elevated levels of loss of heterozygosity (LOH) (22), DNA methylation-based Stemness Scores derived by the Stemness group (DNAss) and RNA-based Stemness scores derived by the Stemness group (RNAss) (23) reflect genomic and transcriptomic instability, and are often correlated with enhanced tumor aggressiveness, poor prognosis, and therapy resistance. Data regarding TMB, MSI, NEO, and LOH were retrived from the TCGA and GDC (https://portal.gdc.cancer.gov/) (24). *ZNF25* expression values were log_2_(x+0.001) transformed, and Pearson correlation analysis was performed to evaluate its associations with TMB, MSI, NEO, and LOH. Results were visualized as lollipop plots using the ggplot2 in R.

The ZNF family is critically involved in regulating tumor stemness, as multiple family members have been shown to correlate with DNAss and RNAss (25). The tumor stemness indices, including the DNAss and RNAss, were derived from mRNA expression and DNA methylation signatures as previously described. Using the Sangerbox platform, we calculated the stemness score of *ZNF25* by integrating its expression data with known stemness-associated features. After excluding fewer than 3 samples, 37 cancer types remained. Pearson correlation coefficients between *ZNF25* expression and DNAss/RNAss were computed and visualized using lollipop plots.

### 2.4 Genetic alterations analysis of *ZNF25* in pan-cancer

The cBioPortal tool was employed to investigate the mutation of *ZNF25* across different cancer types. Initially, we opted for the “TCGA PanCancer Atlas Studies” dataset. Subsequently, we input “*ZNF25*” into the “Query” section, where the tool furnished data about *ZNF25* alteration sites, categories, as well as quantities within both the “Cancer Types Summary” and “Mutations” sections. Besides, the IntOGen (IntOGen-Cancer Mutations Browser) platform was utilized to obtain the distribution of the observed mutations along the protein sequence.

### 2.5 Correlation analysis of DNA methylation and RNA modification for *ZNF25*

The SMART platform (http://www.bioinfo-zs.com/smartapp/) (26) was used to analyze the correlation between *ZNF25* expression and DNA methylation in various cancers in September 2023. Firstly, we inputted “*ZNF25*” into the “Quick Start” module, and subsequently utilized the “CpG-aggregated methylation” module to calculate the pan-cancer *ZNF25* methylation levels and visualized as box plots. The MethSurv web tool (https://biit.cs.ut.ee/methsurv/) (27) was utilized to perform multivariable survival analysis based on *ZNF25* DNA methylation data.

Expression data for the *ZNF25* and 44 genes related to the three RNA modifications types (m1A (10), m5C (13), m6A (21)) were extracted using Sangerbox and log_2_(x + 0.001) transform. Pearson correlation coefficients between *ZNF25* and these RNA modification regulators were calculated.

### 2.6 Correlation analysis of *ZNF25* expression with the TIME

ESTIMATE (Estimation of STromal and Immune cells in MAlignant Tumor tissues using Expression data), a sophisticated scoring system, was used to evaluate the stromal cell presence, and immune cell infiltration in tumor tissue by analyzing expression data (28). The estimate R package and Spearman correlation test was used to calculated stroma, immune, and estimate scores for each patient in each tumor based on gene expression. The results were then visualized using ggplot2, heatmap, ggpubr, and ggExtra R packages.

To analyze the association between *ZNF25* expression and tumor-infiltrating immune cells (TIICs) across pan-cancer, we employed the “Lymphocyte” module of TISIDB (http://cis.hku.hk/TISIDB/search.php) in August 2023 (29), with purity adjustment applied. We used Spearman correlation analysis to explore the relationship between *ZNF25* expression and the infiltration levels of 28 immune cell types, reporting the correlation coefficient as rho (ρ). TIICs included active T cell (ActCD8^+^, ActCD4^+^), Central Memory T cell (TcmCD8^+^, TcmCD4^+^), Effector Memory T cell (TemCD8^+^, TemCD4^+^), Th1, Th2, regulatory T cell (Treg), active B cell (Act B), immature B cell (Imm B), memory B cell (Mem B), natural killer (NK), CD56bright NK (CD56bright), CD56dim NK (CD56dim), Myeloid dendritic cell, natural killer (NK), Myeloid-derived suppressor cell (MDSC), natural killer T cell (NKT), active Dendritic Cell (Act DC), plasmacytoid dendritic cells (pDC), immature Dendritic Cell (iDC), macrophage, eosinophil, mast cell, monocyte, and neutrophil. The results are presented as a heatmap. In addition, we also applied the same method to analyze the relationships of *ZNF25* expression, CNA, and methylation with MHC-related genes (MHCs), respectively. Besides, the correlation between somatic Copy Number Alteration (CNA) of *ZNF25* and immune cell infiltration levels was investigated using TIMER (https://cistrome.shinyapps.io/timer/) in August 2023.

Isocitrate dehydrogenase 1(*IDH1*) mutation is a hallmark molecular event in glioma genesis, occurring in the majority of gliomas, where it serves as a pivotal diagnostic biomarker and an independent and favorable prognostic factor (30, 31). The correlation between *ZNF25* and wild type *IDH1* (*IDH1*-WT), mutated *IDH1 *(*IDH1*-mut) genes expression in GBM and LGG was assessed from “gene correlation” module of the “Cancer Exploration” section in TIMER2.0 database, which integrates various immune prediction methods, calculating significance using the Wilcoxon test. The correlation of *ZNF25* expression with *IDH1*-WT and *IDH1*-mut expression in GBM and LGG was assessed using the TIMER2.0 database. Specifically, we used the “gene correlation” module within the “Cancer Exploration” section, which integrates various immune prediction methods and calculates significance using the Wilcoxon test.

### 2.7 Correlation analysis of *ZNF25* expression with DNA repair genes and T cell exhaustion (Tex)- related genes

DNA repair systems play a critical role in maintaining genome integrity and preventing cancer development. To determine whether *ZNF25* is involved in this process, we used the TIMER 2.0 database to analyze the pan-cancer correlation between *ZNF25* expression and representative markers of DNA repair pathways, including *ADAR*, *ATM*, *BRCA1*, *BRCA2*, *ERCC2*, *ERCC4*, *ERCC5*, *MLH1*, *MLH3*, *MSH2*, *MSH3*, *MSH4*, *MSH5*, *PMS1*, *PMS2*, *XPA*, and *XPC*. The results were visualized as heatmaps.

Tex has a long-term effect on the TIME, which may show an enhanced immune regulatory function by inhibiting the activity of effector immune cells, forming an immune escape environment (32). To explore the association between *ZNF25* expression and Tex cells, we analyzed its relationship with Tex related gene markers (*ARID1A*, *ARID1B*, *CD274*, *CTCF*, *CTNNB1*, *EP300*, *GSK3B*, *HAVCR2*, *IFNAR1*, and *YY1*) using the TIMER 2.0 database.

### 2.8 Cell lines and cultures

Glioma exhibited the most significant associations between *ZNF25* expression and prognosis as well as immune infiltration, with opposite patterns in GBM and LGG. Therefore, we selected two glioma cell models (A172 and U87-MG) for subsequent experiments, including knockdown and phenotypic assays. Glioma cells U87-MG were obtained from the American Type Culture Collection (ATCC, Manassas, USA), and A172 were purchased from Xiamen Immocell Biotechnology Co. Ltd. Cells were cultured in DMEM medium (Gibco, ThermoFisher, China) supplemented with 100U/ml penicillin-streptomycin (HyCloneTM, Utah, USA) and 10% fetal bovine serum (FBS, ExCell, China). All cultures were maintained at 37 °C in a humidified incubator with 5% CO_2_.

### 2.9 siRNA reverse transfection assay

To evaluate the knockdown efficiency of the three siRNAs (GenePharma, Shanghai, China), reverse transfection was performed in 6-well plates. The siRNAs panel included three siRNAs targeting *ZNF25* and a non-targeting negative control siRNA (siNC), with three biological replicates for each condition. The transfection reagent (FECT1, Dharmacon Reagents, USA) was gently mixed with Opti-MEM, followed by the addition of the siRNAs, and incubated at room temperature for 30 minutes to allow complex formation. U87-MG and A172 cells were seeded into the 6-well plates at a density of 3 × 10^5^ cells per well, after which the transfection complexes were evenly added. The final concentration of siRNA in the culture medium was 25 nM. After 72 hours of incubation at 37 °C, the knockdown efficiency of siRNA was assessed by RT-qPCR, and RNA-seq analysis was subsequently performed using U87-MG.

The sequence of *ZNF25* siRNAs are as follows:

*ZNF25* siRNA-1: ACAUCCUUAUACAGAGUCCTT;
*ZNF25* siRNA-2: UCUAGAUCAUGAAUGCUCCTT;
*ZNF25* siRNA-3: UGAUGUACUAUGAGGGCAGTT;
NC siRNA: ACGUGACACGUUCGGADAATT.

### 2.10 Real-time fluorescence quantitative PCR

Six 10 cm dishes were used for the knockdown assay, with 7× 10^5^ U87-MG cells seeded per dish. The cells were divided into two groups: siNC and si*ZNF25*, with three biological replicates per group (one dish per replicate). The si*ZNF25* pool consisted of three siRNAs (si*ZNF25*-1, si*ZNF25*-2, and si*ZNF25-*3; GenePharma, Shanghai, China). Total mRNA was extracted using Trizol (Invitrogen, USA) according to the manufacturer’s instructions. The extracted total mRNA was reverse-transcribed into cDNA using the HiScript II Q RT SuperMix for qPCR kit (Vazyme, China), followed by RT-qPCR amplification using ChamQ Universal SYBR qPCR Master Mix (Vazyme, China), with β-actin as the internal reference gene. Relative gene expression was calculated using the 2^(-ΔΔCT) method. The primer sequences used are listed as follows:

*ZNF25*-Forward primer 5’-3’: CCTGGGGCTGCCAGCTAAGGT;
*ZNF25*-Reverse primer 5’-3’: CAGGGAAGCCCCGATGTGGAA.
β-actin-Forward primer 5’-3’: CATGTACGTTGCTATCCAGGC;
β-actin -Reverse primer 5’-3’: CTCCTTAATGTCACGCACGAT.

### 2.11 Western blot assays

U87-MG and A172 cells were transfected as described in Section 2.10. Total protein was extracted from both the si*ZNF25* groups and siNC group using RIPA lysis buffer. Protein quantification was performed using the Bicinchoninic Acid (BCA) assay. Samples were denatured by heating at 100 °C for 10 minutes in a metal bath, briefly cooled, and centrifuged prior to loading. Equal amounts of protein were separated by Sodium Dodecyl Sulfate-Polyacrylamide Gel Electrophoresis (SDS-PAGE), and transferred onto PVDF membranes, followed by blocking with 5% non-fat milk. The membranes were then incubated overnight at 4 °C with primary antibodies against the target protein and GAPDH (loading control), followed by incubation with an HRP-conjugated secondary anti-bodies. Protein bands were visualized using an enhanced chemiluminescence (ECL) substrate, and band intensities were quantified using ImageJ software. Relative protein expression levels were normalized to GAPDH, and statistical significance was assessed using an unpaired t-test based on at least three independent experiments.

### 2.12 Cell viability assays

U87-MG and A172 cells were respectively seeded into 96-well plates at a density of 5× 10^3^ cells per well, and divided into control and knockdown groups. Cells were transfected as described in Section 2.10, and incubated at 37 °C for 72h. The experiment was independently repeated three times. Subsequently, 10μl of Cell Counting Kit-8 (CCK-8) reagent (BaoGuang Bio, China) was added to each well. After an additional 2 hours of incubation, the absorbance at 450 nm was measured using a microplate reader. Cell viability was calculated by comparing the absorbance values between the knockdown and control groups. Absorbance was measured at 450 nm using a spectrophotometer. The results of the CCK-8 assay were presented as bar charts generated using GraphPad Prism.

### 2.13 Wound healing assays

U87-MG and A172 cells were respectively seeded into 96-well plates at a concentration of 5× 10^3^ cells/well and reverse transfected as described in Section 2.10. Upon reaching approximately 90% confluence, a precise wound was created in each well using a 96 wells cell wound maker (Sartorius, IncuCyte^®^).The plates were then transferred to a live-cell analysis system (Sartorius, IncuCyte^®^ SX1) for continuous incubation and automated image acquisition. The system captured phase-contrast images at the predefined time points of 0, 12, 24, and 36 hours post-scratching. Wound closure was quantified using the integrated software by measuring the wound width or confluence. Wound closure efficiency was quantified and statistically analyzed at the 36-hour time point, with six independent experimental replicates. Quantitative results were presented as bar charts generated using GraphPad Prism.

### 2.14 RNA-seq experiments

RNA-seq experiment was conducted on U87-MG cell samples treated with siNC or si*ZNF25*. First, total RNA was extracted using Trizol reagent. Second, RNA quantity and purity were detected by Bioanalyzer 2100 and RNA 6000 Nano Kit (Agilent, USA). Only RNA samples with an RNA integrity number (RIN) >7.0 were used for library construction. Then, the sequencing libraries were prepared and subjected to transcriptome sequencing on the Illumina NovaseqTM 6000 sequence platform (LC-Bio Technologies (Hangzhou) Co, Ltd, China), with a minimum depth of 20 million reads per sample. All libraries passed stringent quality control criteria, including a Q30 score > 90%, ensuring superior data accuracy for reliable results. The detected gene counts were distributed uniformly across all samples without obvious outliers, supporting data reliability for downstream analysis. Illumina sequence FASTQ reads were aligned to the human reference genome (GRCh38) using HiSat2 (25). Gene expression levels were quantified and differential expression analysis was performed using DESeq2. DEGs were identified with cut-offs of adjusted P-value (padj) < 0.05 and log_2_ fold-change (log_2_FC) > 1.

### 2.15 Gene enrichment analysis

To identify the biological pathways and key genes affected by *ZNF25* knockdown, we performed functional enrichment analysis on the DEGs obtained from RNA-seq following *ZNF25* silencing. The volcano plot illustrating the DEGs was generated using the Sangerbox platform. DAVID (https://davidbioinformatics.nih.gov/) (33) was employed for conducting Gene Ontology (GO) and Kyoto Encyclopedia of Genes and Genomes (KEGG) enrichment analysis, and identified the top 10 significantly pathways. The results were visualized by ggplot R package.

### 2.16 Statistical analysis

All statistical analyses and visualizations were performed using R software and GraphPad Prism 9.5.0. The correlation between two groups is calculated using Pearson or Spearman correlation analysis, and for significant differences in prognosis analysis, the log-rank test is employed. Statistical analysis of RT-qPCR data is performed using Student’s t-test. Statistical significance was defined as P < 0.05 (*), P < 0.01 (**), P < 0.001 (***), and P < 0.0001 (****).

## 3 Results

### 3.1 *ZNF25* is differentially expressed across cancers and associated with patient prognosis

**Figure 1:**
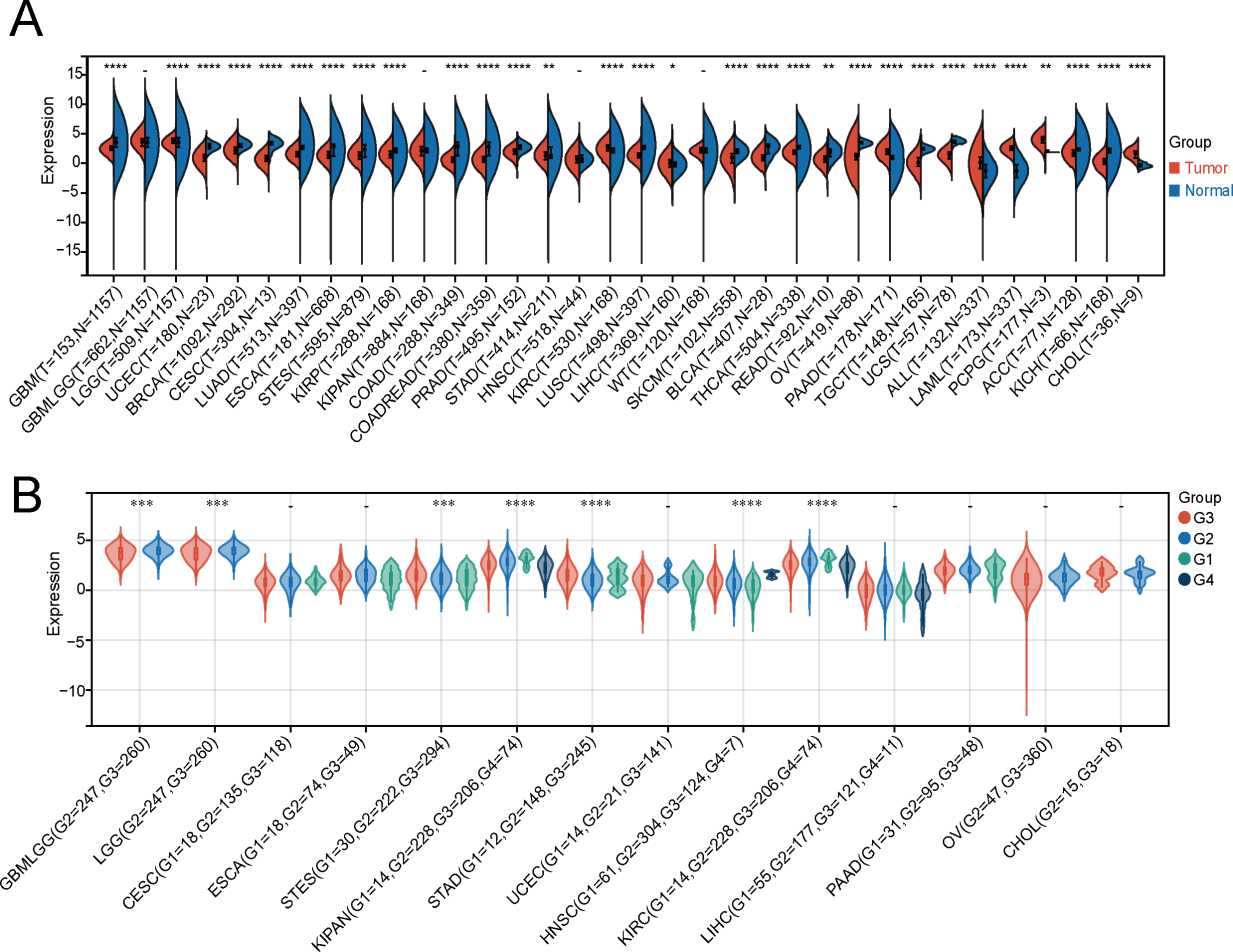
*ZNF25* exhibited significantly different expression across multiple cancer types. **(A)** *ZNF25* exhibited low expression levels in 22 types of tumors and was highly expressed in 8 types of tumors. **(B)** *ZNF25* displayed significant different expression levels in different tumor grade of in 14 types of cancers and normal tissues from the TCGA and GTEx database. Differences were assessed using the Wilcoxon rank-sum test (for unpaired samples) and the Wilcoxon signed-rank test (for paired samples). The resulting P-values were adjusted using p.adjust function in R 4.5.0. (* adjust P <0.05, ** adjust P < 0.01, *** adjust P < 0.001, **** adjust P < 0.0001).

Pan-cancer analysis revealed that *ZNF25* expression was significantly dysregulated across multiple malignancies. Specifically, *ZNF25* was significantly downregulated in 22 cancer types, including GBM, BRCA, LUAD, COAD, and BLCA, while upregulated in 8 cancers, including LGG, KIRC, LIHC and PAAD (**Figure 1A**). Additionally, *ZNF25* exhibited opposite expression patterns in glioma, with low expression in GBM and high expression in LGG. Further analysis showed significant differences in *ZNF25* expression across tumor grades in GBMLGG, LGG, STES, KIPAN, STAD, HNSC, and KIRC (**Figure 1B**).

**Figure 2:**
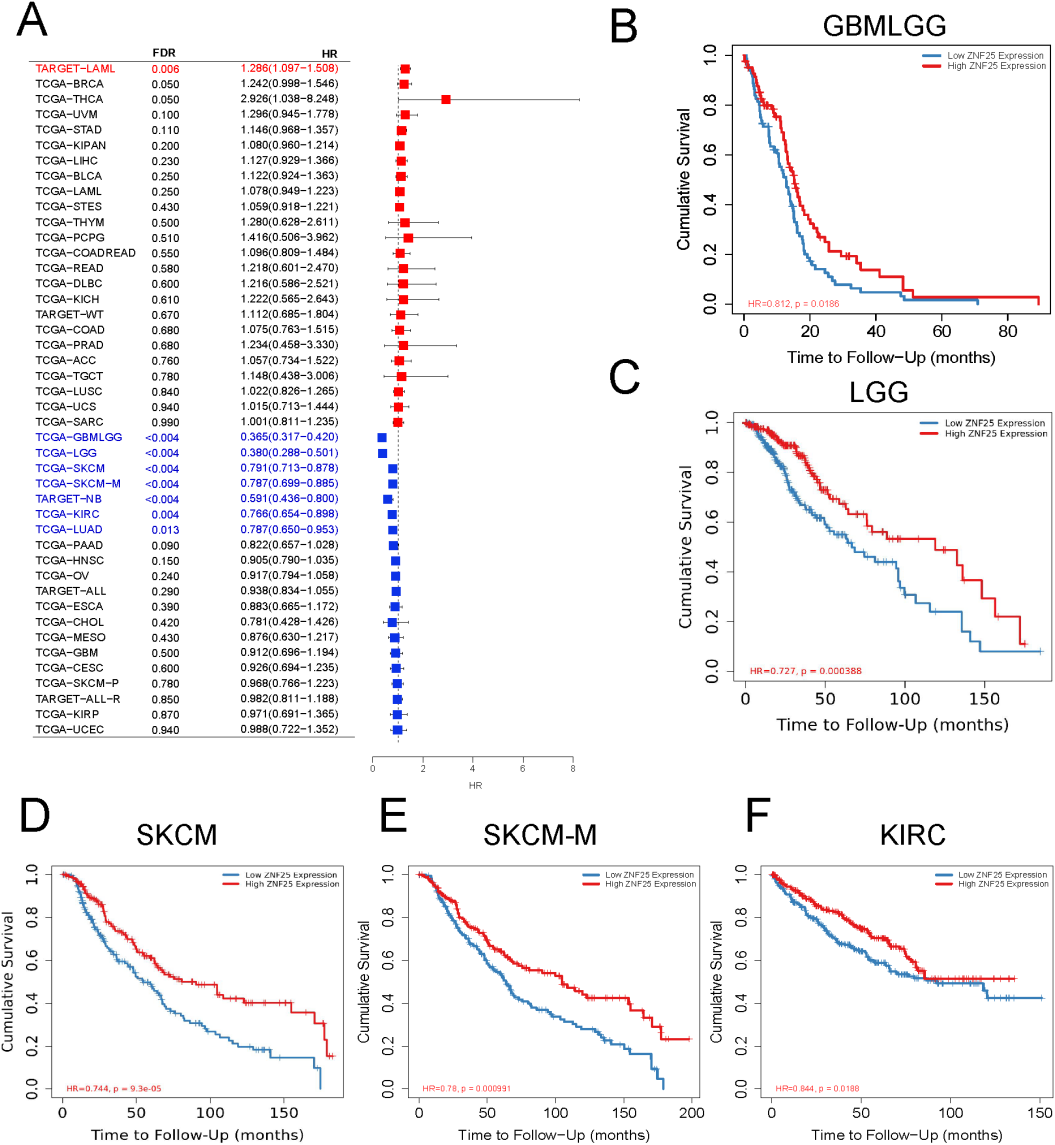
The survival prognosis analysis of *ZNF25* expression in multiple cancers. **(A)** *ZNF25*expression correlated with a favorable prognosis of OS in 7 types of cancer patients and an unfavorable prognosis of OS in 1 type of cancer patients. Forest plot depicting the survival analysis results of *ZNF25* expression on OS in pan-cancer (FDR < 0.05). **(B–F)** Results of Kaplan-Meier analysis of significance between *ZNF25* expression and OS (P < 0.05). High expression of *ZNF25* was correlated with a favorable prognosis of OS in GBMLGG, LGG, SKCM, SKCM-M, and KIRC.

Survival analysis showed that *ZNF25* expression is significantly associated with patient outcomes in a cancer-type-specific manner. *ZNF25* exhibited a favorable prognostic factor in glioma, with hazard ratios (HR) of 0.365 in GBMLGG and 0.380 in LGG (both FDR<0.004), indicating that higher expression predicted better survival (**Figure 2A**). Similar associations were observed in SKCM, SKCM-M, KIRC, and LUAD. Consistent trends were observed for DSS and DFI, particularly in glioma (**Supplementary Figures 1A, B**). Kaplan-Meier curves showed low *ZNF25* expression correlated with poor prognosis in GBMLGG, LGG, SKCM, SKCM-M, and KIRC (**Figures 2B–F**). These findings support *ZNF25* as a context-dependent prognostic biomarker with particular relevance in glioma.

### 3.2 *ZNF25* correlates with tumor genomic features and stemness

**Figure 3:**
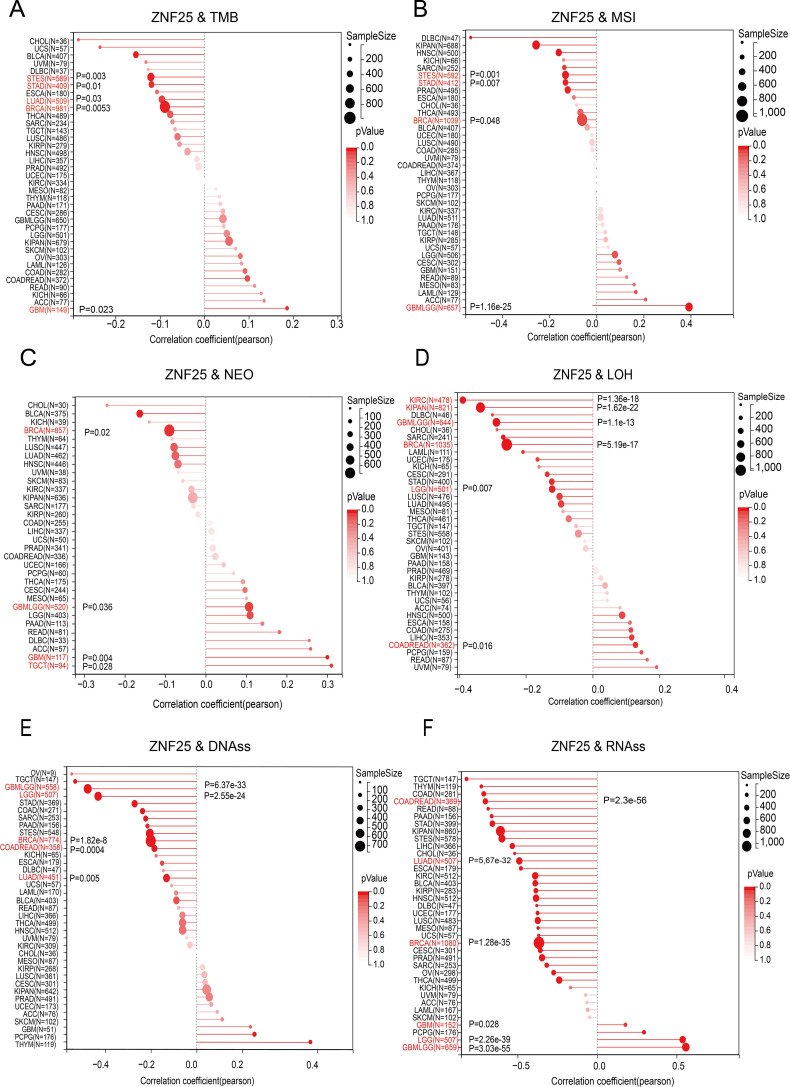
The expression of *ZNF25* is significantly associated with tumor heterogeneity and tumor stemness in multiple types of cancer. **(A)** Lollipop plots of the correlation between *ZNF25* expression and TMB. **(B)** Lollipop plots of the correlation between *ZNF25* expression and MSI. **(C)** Lollipop plots of the correlation between *ZNF25* expression and NEO. **(D)** Lollipop plots of the correlation between *ZNF25* expression and LOH. **(E)** Lollipop plots of the correlation between *ZNF25* expression and DNAss. **(F)** Lollipop plots of the correlation between *ZNF25* expression and RNAss.

To explore the genomic relevance of *ZNF25*, we analyzed its association with tumor heterogeneity indicators. *ZNF25* expression showed significant correlations with TMB, MSI, NEO, and LOH across multiple caners, including a positive association with TMB and neoantigen burden in GBM (**Figures 3A–D**). For example, *ZNF25* was positively associated with TMB and NEO in GBM and GBMLGG, while negatively correlated in cancers such as BRCA and LUAD. Furthermore, *ZNF25* was significantly associated with tumor stemness indices (DNAss and RNAss) in a wide range of cancers (**Figures 3E, F**). In LGG and GBMLGG, *ZNF25* showed significantly negative correlated with DNAss and positive correlated with RNAsss, indicating its potential involvement in tumor dedifferentiation and progression.

### 3.3 *ZNF25* genetic alterations in pan-cancer

**Figure 4:**
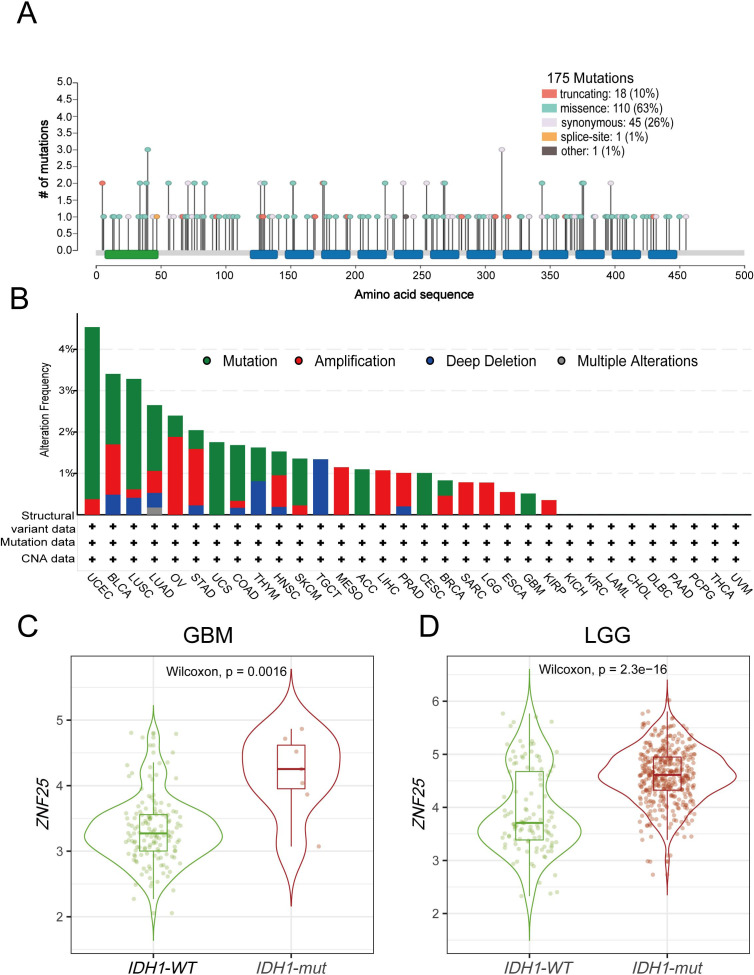
Genetic alterations of *ZNF25* in pan-cancer. **(A)** Genetic alterations of *ZNF25* protein structural domains. **(B)** Mutation types and frequency of *ZNF25* gene in pan-cancer. **(C, D)** The upregulation of *ZNF25* in *IDH1-*mut type of GBM and LGG.

Analysis of 25,698 tumor samples revealed that *ZNF25* alterations were relatively infrequent, with missense mutations being the predominant type (**Figure 4A**). Higher mutation frequencies were observed in UCEC (4.54%), BLCA (3.41%), and LUAD (3.29%) (**Figure 4B**). *ZNF25* expression was also positively correlated with multiple DNA repair-related genes in pan-cancer, including ADAR and ATM (**Supplementary Figure 2A**), raising the possibility that *ZNF25* may be functionally linked to genomic stability maintenance.

In glioma, *ZNF25* expression was significantly higher in *IDH1*-mut types compared to *IDH1*-WT case in both GBM and LGG (**Figures 4C, D**). The *IDH1*-mut molecular subtype is independently associated with a more favorable prognosis. These findings suggest that *ZNF25* may participate in the oncogenic pathway related to *IDH1* mutation and could serve as a potential molecular marker to distinguish between *IDH1*-mut and *IDH1*-WT gliomas.

### 3.4 *ZNF 25* is closely associated with the tumor immune infiltration, particularly in glioma

*ZNF25* expression was positively correlated with Tex genes in almost all cancer types (**Supplementary Figure 2B**), indicating that *ZNF25* may be involved in the immune escape microenvironment.

**Figure 5:**
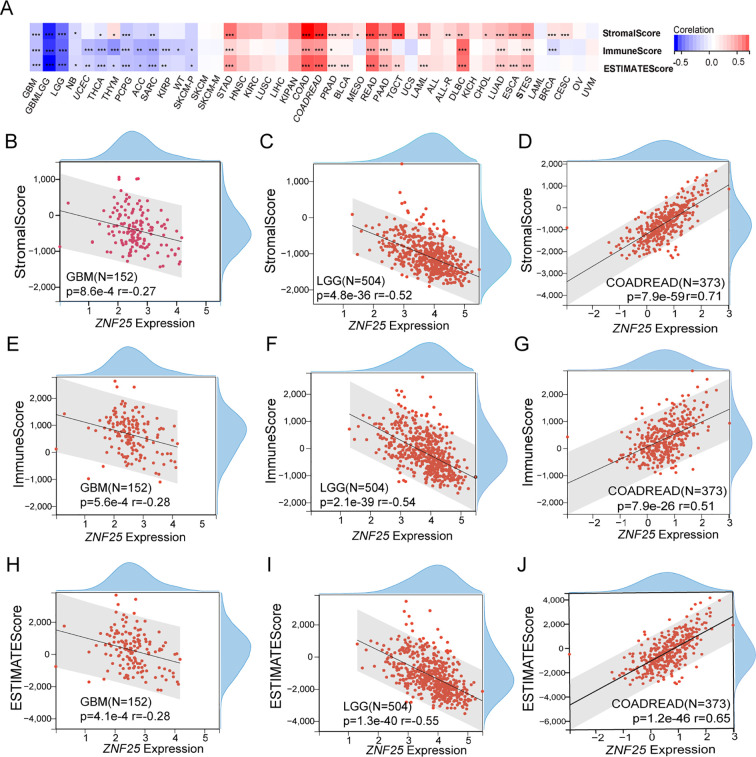
ZNF25 expression is associated with stromal scores, immune scores, and estimate scores in the TIME across various cancer types. **(A)** Heatmap showing the correlation between *ZNF25* in pan-cancer and StromalScore, ImmuneScore, and ESTIMATEScore, red represents positive correlation, and blue represents negative correlation. **(B–D)** Specific correlation plots between *ZNF25* expression in GBM, LGG, COADREAD and StromalScore. **(E–G)** Specific correlation plots between *ZNF25* expression in GBM, LGG, COADREAD and ImmuneScore. **(H–J)** Specific correlation plots between *ZNF25* expression in GBM, LGG, COADREAD and ESTIMATEScore. (* P <0.05, ** P < 0.01, *** P < 0.001).

*ZNF25* expression was significantly correlated with immune infiltration in 25 cancer types (**Figure 5A**). Positive correlations ESTIMATEScore were observed in 13 cancer types, including LUAD, and COAD, whereas negative correlations were prominent in glioma (GBM, GBMLGG, and LGG). Correlation analyses between *ZNF25* expression and StromalScore, ImmuneScore, and ESTIMATEScore across multiple cancer types, including GBM, LGG, COADREAD, KIPAN, COAD, and PAAD, further supported these findings (**Figures 5B–J**).

**Figure 6:**
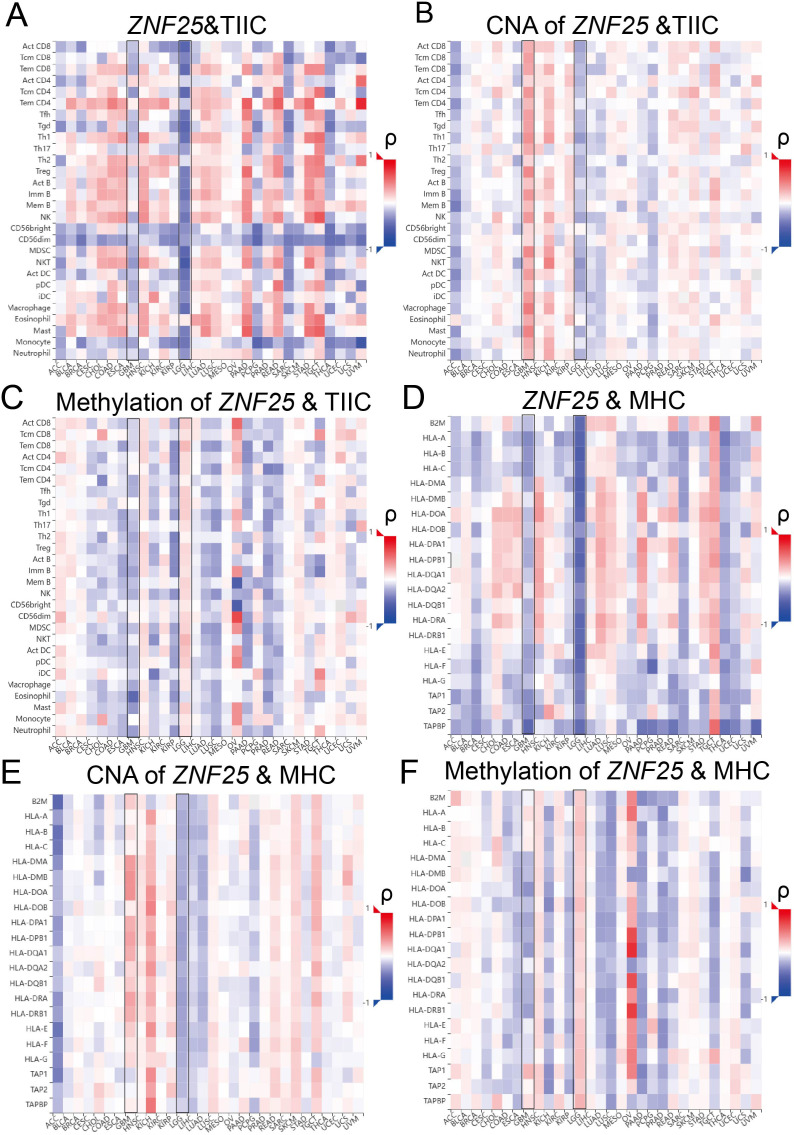
The relationship between *ZNF25* expression and TIICs, MHCs in pan-cancer. **(A)** Association between *ZNF25* expression and TIIC across 30 types of cancer. **(B)** Association between CNA of *ZNF25* expression and TIICs across 30 types of cancer. **(C)** Association between *ZNF25* methylation and TIICs across 30 types of cancer. **(D)** Association between *ZNF25* expression and MHCs across 30 types of cancer. **(E)** Association between CNA of *ZNF25* expression and MHCs across 30 types of cancer. **(F)** Association between *ZNF25* methylation and MHCs across 30 types of cancer. The red color indicates significant positive correlation and the blue color indicates significant negative correlation.

Analysis of tumor-infiltration immune cells (TIICs) revealed that *ZNF25* expression was negatively associated with most infiltration immune cells both in GBM and LGG (**Figure 6A**). Intriguingly, the correlation between *ZNF25* methylation and TIICs was positive in GBM but negative in LGG. In contrast, the correlation between CNA of *ZNF25* and TIICs was negative in GBM but positive in LGG (**Figures 6B, C**). Furthermore, *ZNF25* expression was negatively associated with MHCs both in GBM and LGG, but positively correlated in TGCT (**Figure 6D**). While the correlation between CNA of *ZNF25* and MHCs is positive in GBM but negative in LGG, and the correlation between *ZNF25* methylation and MHCs is almost negative in GBM but positive in LGG (**Figures 6E, F**).

Given the growing body of evidence on the role of RNA modification in tumorigenesis, our pan-cancer investigation demonstrated a significant positive correlation between *ZNF25* expression and the majority of RNA modification associated regulators, encompassing writers, readers, and erasers (**Supplementary Figure 3A**).Besides, arm-level deletions of *ZNF25* were significantly associated with immune cell infiltration in LGG, including B cell, CD8^+^ T cell, CD4^+^ T cell, macrophage, neutrophil, and dendritic cell. In contrast, in GBM, arm-level deletions of *ZNF25* were only significantly correlated with Macrophage, Neutrophil, and Dendritic cells (**Supplementary Figure 3B**).

Collectively, these results indicated that *ZNF25* may serve as a factor that dynamically evolves during glioma progression, with its distinct molecular forms (expression, methylation, and CNA) playing different roles in the immune microenvironment of glioma.

### 3.5 Correlation between *ZNF25* DNA methylation and patient survival

**Figure 7:**
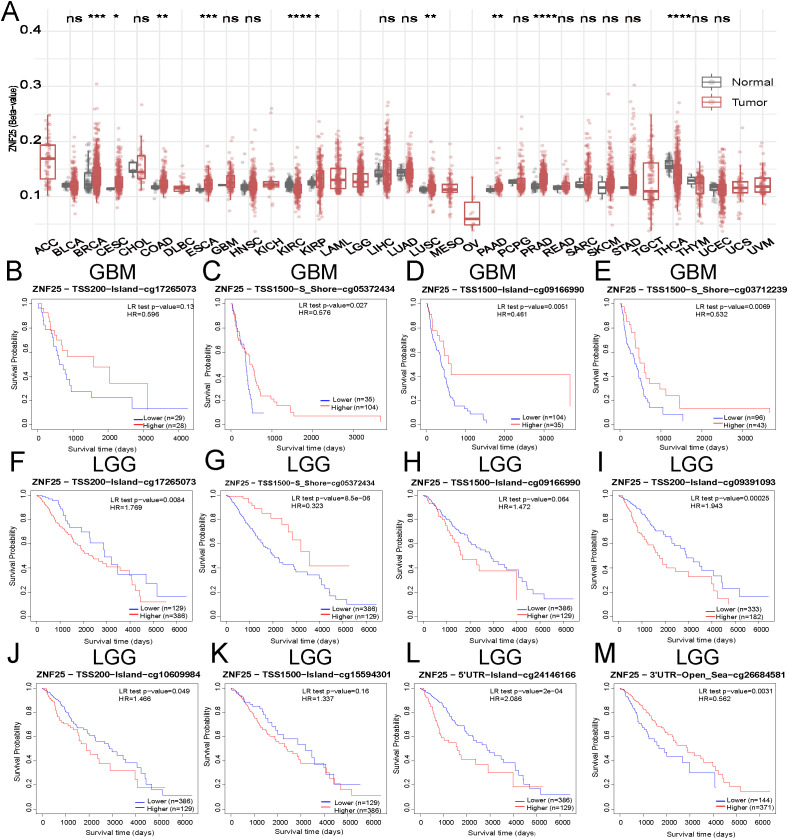
The correlation between DNA methylation sites of *ZNF25* and their correlation with survival probability analysis. **(A)** Promoter methylation levels of *ZNF25* promotor in pan-cancer. **(B–E)** Results of Kaplan-Meier analysis of significance between *ZNF25* expression and DNA methylation in GBM. **(F–M)** Kaplan-Meier analysis of significance between *ZNF25* expression and DNA methylation in LGG. (* P <0.05, ** P< 0.01, *** P < 0.001, **** P < 0.0001).

DNA methylation analysis revealed significant tumor-specific alterations in *ZNF25* promoter methylation across pan-cancer (**Figure 7A**). In glioma, different *ZNF25* methylation sites showed distinct or even opposite prognostic effects. For example, methylation at TSS200 sites (e.g., cg10265073) was negatively correlated with survival, whereas certain TSS1500 sites were positively correlated with survival (**Figures 7B–L**). Additionally, cg09166990 (TSS1500 island) and cg03712239 (TSS1500 island-S-Shore) exhibited a positive correlation with survival in GBM (**Figures 7D, E**), while cg09166990 (TSS1500 island) displayed a negative correlation with survival in LGG (**Figure 7H**). In LGG, methylation at cg09391093 and cg10609984 (TSS200 island) were negatively correlated with survival probability (**Figures 7I, J**), and the methylation of TSS1500-island-cg15594301 and 5’ Untranslated Region (5’UTR)-island-cg24146166 of *ZNF25* is negatively related with survival (**Figures 7K, L**). Interestingly, methylation at cg26684581 (3’ Untranslated Region (3’UTR)-Open-sea) was positively correlated with survival in LGG (**Figure 7M**).

### 3.6 *ZNF25* knockdown suppress proliferation and migration in glioma cells

**Figure 8:**
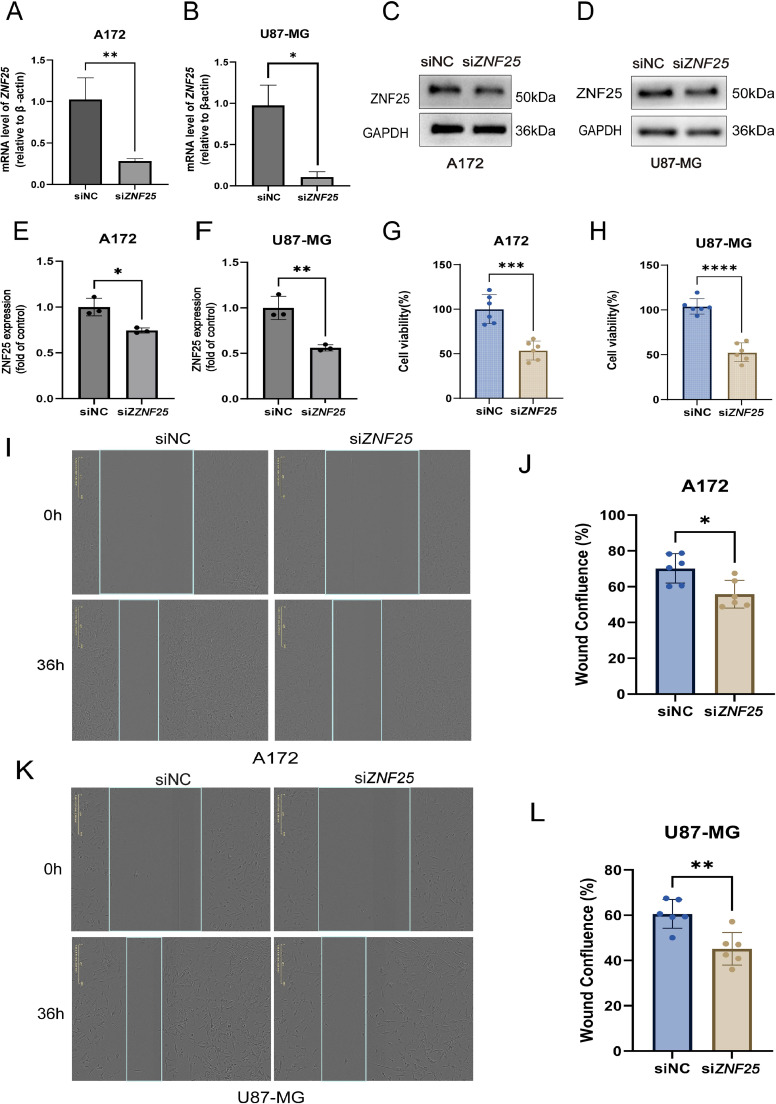
*ZNF25* knockdown inhibits proliferation and migration of A172 and U87-MG Cells. **(A)** The silencing efficiency of siRNAs for *ZNF25* knockdown in A172 cells detected by RT-qPCR. **(B)** The silencing efficiency of siRNAs for *ZNF25* knockdown in U87-MG cells detected by RT-qPCR. **(C)** ZNF25 protein levels in A172 cells were analyzed by Western blotting at 72 hours post-transfection with si*ZNF25* and siNC. GAPDH was used as a loading control. **(D)** ZNF25 protein levels in U87-MG cells were analyzed by Western blotting at 72 hours post-transfection with si*ZNF25* and siNC. GAPDH was used as a loading control. **(E)** Bar graph showing the quantification of protein expression in A172 cells. The band intensities from Western blot analysis were quantified using ImageJ software. Data are presented as mean ± SD (n=3 independent experiment, Student’s t-test). **(F)** Bar graph showing the quantification of protein expression in U87-MG cells. The band intensities from Western blot analysis were quantified using ImageJ software. Data are presented as mean ± SD (n=3 independent experiment, Student’s t-test). **(G–H)** Cell viability was determined by CCK-8 assay in A172 and U87-MG cells after transfection with si*ZNF25* and siNC. Data are presented as the mean ± SD of at least three independent experiments compared with the siNC group at the corresponding time point. **(I)** Representative microscopic images of the wound area at 36h after scratching in A172 cells transfected with si*ZNF25* and siNC. Scale bar, 400 μm. **(J)** Quantitative analysis of the wound closure rate of A172 cells at 36h post-transfection with si*ZNF25* and siNC. **(K)** Representative microscopic images of the wound area at 36h after scratching in U87-MG cells transfected with si*ZNF25* and siNC. Scale bar, 400μm. **(L)** Quantitative analysis of wound closure rate in U87-MG cells at 36h post-transfection, with si*ZNF25* and siNC. Data are shown as mean ± SD from n = 6 independent experiments. Statistical significance was determined by Student’s t-test. (* P <0.05, ** P< 0.01, *** P < 0.001, **** P < 0.0001).

To further investigate the functional role of *ZNF25*, its expression was silenced in U87-MG and A172 glioma cell lines using small interfering RNAs (si*ZNF25*), with a non-targeting siRNA (siNC) serving as the control. Efficient knockdown at the transcriptional level was confirmed by both mRNA and protein levels (**Figures 8A–F**). CCK-8 assays demonstrated that *ZNF25* silencing significantly impaired cell proliferation in both A172 and U87-MG cells, and reduced their migratory capacity (**Figures 8G, H**). Wound healing assay revealed that at 36 hours post-wounding, the percentage of wound closure in *ZNF25*-knockdown A172 and U87-MG cell lines was significantly lower than that in the control group (**Figures 8I–L**). Collectively, these findings indicate that *ZNF25* is essential for maintaining the proliferative and migratory potential of glioma cells.

### 3.7 Transcriptomic profiling reveals PI3K-AKT signaling as a key pathway regulated by *ZNF25*

**Figure 9:**
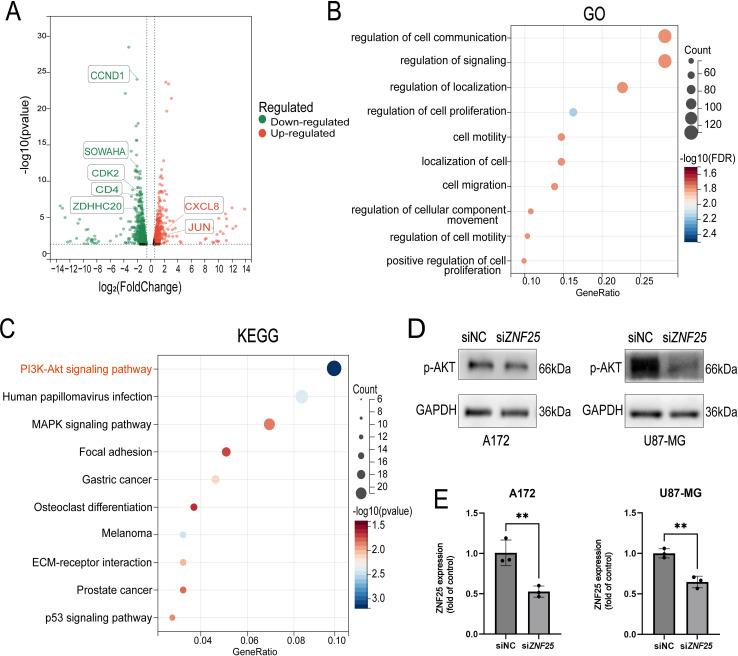
Functional enrichment analysis and molecular verification of *ZNF25* knockdown. **(A)** The volcano plot visualized all identified DEGs in U87-MG cells, which were screened by the criteria of adjusted P-value (padj) < 0.05 and ∣log_2_FC∣>1. **(B)** Top 10 enriched GO terms across all categories. **(C)** Top 10 enriched KEGG pathways. **(D)** Western blot analysis of p-AKT protein levels in A172 and U87-MG cells at 72h after transfection with si*ZNF25* and siNC. GAPDH was used as a loading control. **(E)** Bar graph showing the quantification of protein expression in U87-MG cells corresponding to **(D)**. Data are presented as mean ± SD (n = 3 independent experiment, Student’s t-test). (* P < 0.05, ** P < 0.01).

RNA-seq was conducted on U87-MG cells transfected with si*ZNF25* and siNC. Compared with the siNC group, *ZNF25* knockdown resulted in the significant upregulation of 192 genes and downregulation of 463 genes, as illustrated in the volcano plot (**Figure 9A**). GO enrichment showed involvement in regulation of cell communication, signaling regulation, and regulation of cell proliferation (**Figure 9B**). KEGG analysis revealed that DEGs were significant enrichment in cancer-related pathways, particularly the PI3K-AKT signaling pathway (**Figure 9C**).

Western blot analysis further confirmed that *ZNF25* knockdown reduced p-AKT protein levels both in A172 and U87-MG cells, indicating that *ZNF25* knockdown can inhibit PI3K-AKT signaling pathway (**Figures 9D, E**). Collectively, *ZNF25* silencing suppressed the PI3K-AKT signaling pathway. These results suggest that *ZNF25* promotes glioma progression through activation of PI3K-AKT signaling.

## 4 Discussion

Accumulating evidence has confirmed that KRAB-ZNF family members have been reported to regulate tumor immunity through epigenetic regulatory mechanisms, including DNA methylation, histone modification, and chromatin architecture remodeling. Representative examples such as *ZNF384* (34) and *ZNF423* (35) have been shown to modulate immune cell differentiation and anti-tumor immune responses through epigenetic pathways (36, 37). Nevertheless, the specific biological function and molecular mechanism of *ZNF25* in glioma remain poorly clarified, which highlights the necessity of our present study.

In this work, we first performed comprehensive pan-cancer analysis and found that *ZNF25* exhibited distinct expression patterns across different tumor types. Aberrant *ZNF25* expression was closely related to tumor grade and poor prognosis in glioma. Functional experiments further confirmed that *ZNF25* knockdown significantly inhibited the proliferation and migration of glioma cells. Combined enrichment analysis and molecular verification demonstrated that *ZNF25* participates in glioma progression by positively regulating the PI3K-AKT signaling pathway. In brief, our results reveal that *ZNF25* acts as a key oncogenic factor and promising clinical biomarker in glioma.

*ZNF25* expression is tightly linked to tumor heterogeneity, cancer stemness, genomic instability and Tex across multiple cancer types. Moreover, *ZNF25* was markedly correlated with the infiltration of tumor immune infiltrating cells and molecule expression. Notably, *ZNF25* displayed opposite expression and immune-related characteristics between LGG and GBM, indicating that *ZNF25* exerts context-dependent and subtype-specific regulatory roles in the TIME.

Our study establishes a critical association of *ZNF25* with PI3K-AKT pathway. PI3K-AKT pathway is frequently dysregulated in cancer, and is closely associated with tumor initiation, invasion, and drug resistance (38, 39). In addition, *ZNF25* is located on chromosome 10, and the LOH of chromosome 10, one of the most common genetic events in *IDH1*-WT GBM, is observed more frequently in GBM than LGG (40, 41).

*ZNF25* represents a promising biomarker and therapeutic target for glioma. Moreover, dynamic alterations in its expression constitute a critical molecular event indicative of the malignant progression of glioma. Future research will verify its prognostic value in larger sample cohorts and deeply explore the differential molecular mechanisms by which it functions in LGG and GBM. Besides, elucidating the specific molecular mechanisms of *ZNF25* holds great promise for advancing understanding of cancer biology and refining treatment strategies for gliomas and other malignancies.

This study has some limitations, despite systematically uncovering the correlation between *ZNF25* and various types of cancer, and finding its potential biological processes and signaling pathways that may be involved in regulation. Much of our findings derived from the TCGA pan-cancer atlas, etc. Potential selection bias in these database cohort and unresolved technical batch effects may influence the analysis results. Furthermore, most of our immune-related findings are based on associative bioinformatic analyses, they reveal correlations rather than prove causation. Therefore, the biological functions and molecular mechanisms of *ZNF25* in tumors warrant further investigation, especially in TIME. Besides, it is deserving further investigation the other members of the KRAB-ZNF family are involved in cancer, particularly in gliomas. Understanding these complex regulatory networks could provide insights into tumor development mechanisms and potential treatment approaches.

## Data Availability

The datasets presented in this study can be found in online repositories. The RNA-seq data generated and analyzed in this study have been deposited in the Gene Expression Omnibus (GEO) repository under accession number GSE272124.
