# Supplementary material for: ZNF25 as an immunotherapy target: pan-cancer biomarker potential and mechanistic exploration in glioma
Source: Front Oncol. 2026 Jun 2;16:1631383. doi: 10.3389/fonc.2026.1631383 (PMC13268941; doi:10.3389/fonc.2026.1631383)
Supplement: Supplementary file 1 [file DataSheet1.docx]

**Figure S1**


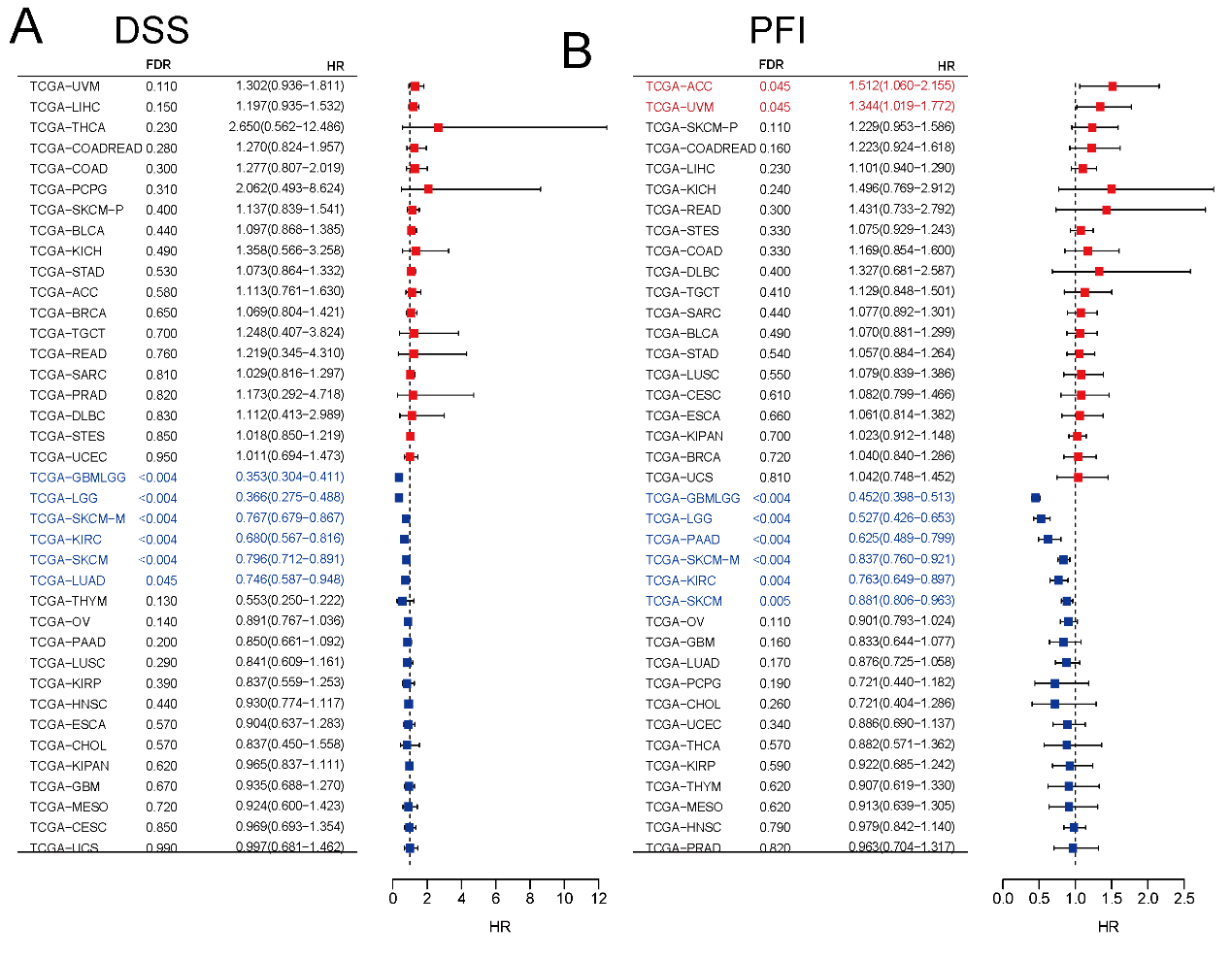


**Figure S1**. Forest plot depicting the survival analysis results of *ZNF25* expression on DSS and PFI in pan- cancer. (A) Low expression of *ZNF25* is significantly correlated with an unfavorable prognosis of DSS in 6 types of cancer patients. (B) Low expression of *ZNF25* is significant correlated with an unfavorable prognosis of PFI in 6 types of cancer patients, with LGG and GBMLGG being common to both analyses. (FDR < 0.05)

**Figure S2**


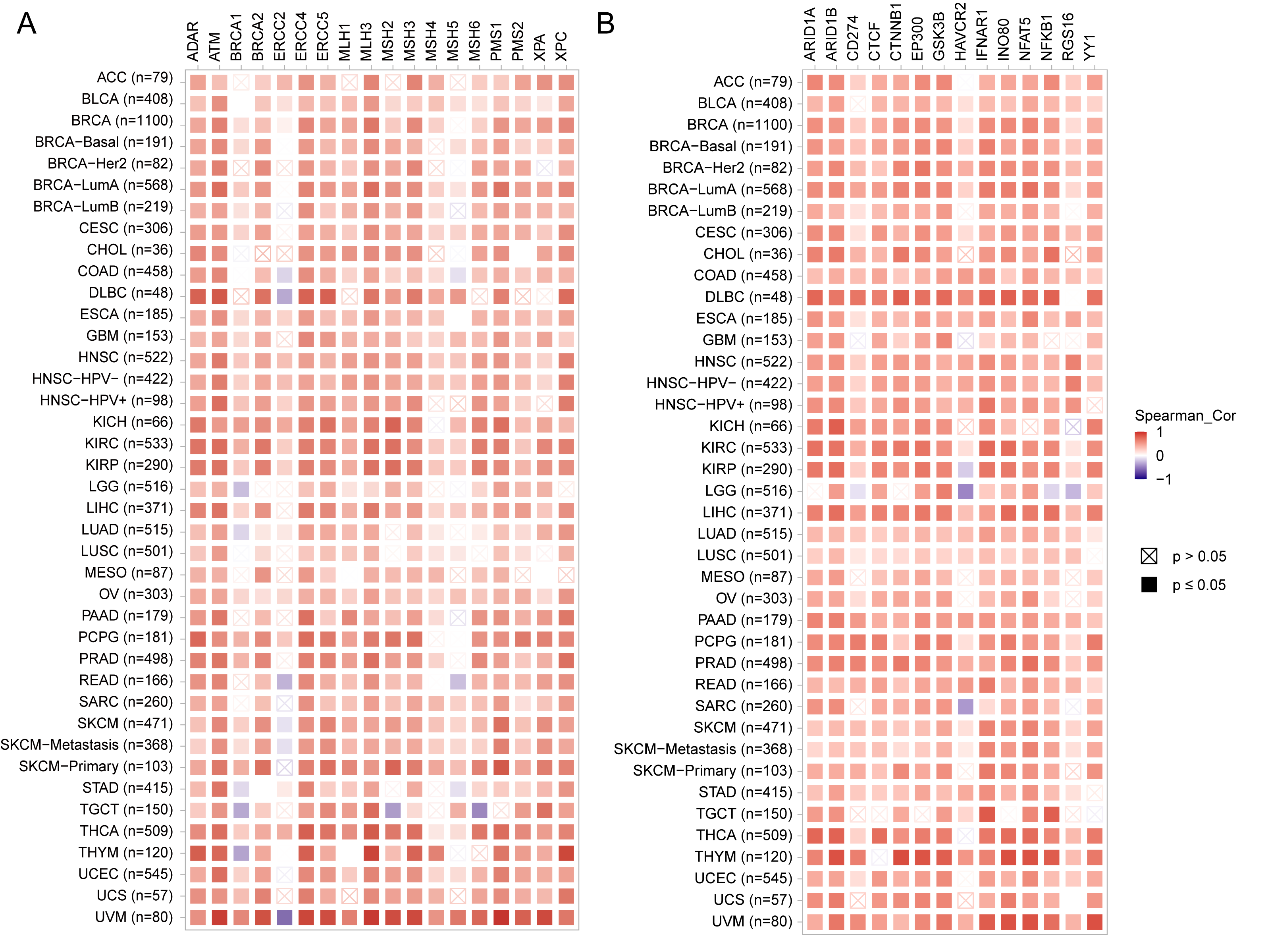


**Figure S2**. The correlation between *ZNF25* and DNA repair genes and Tex marker genes. (A) *ZNF25* significantly correlates with most DNA repair genes in almost all cancer types. (B) *ZNF25* significantly correlates with Tex marker genes in almost all cancer types.

**Figure S3**


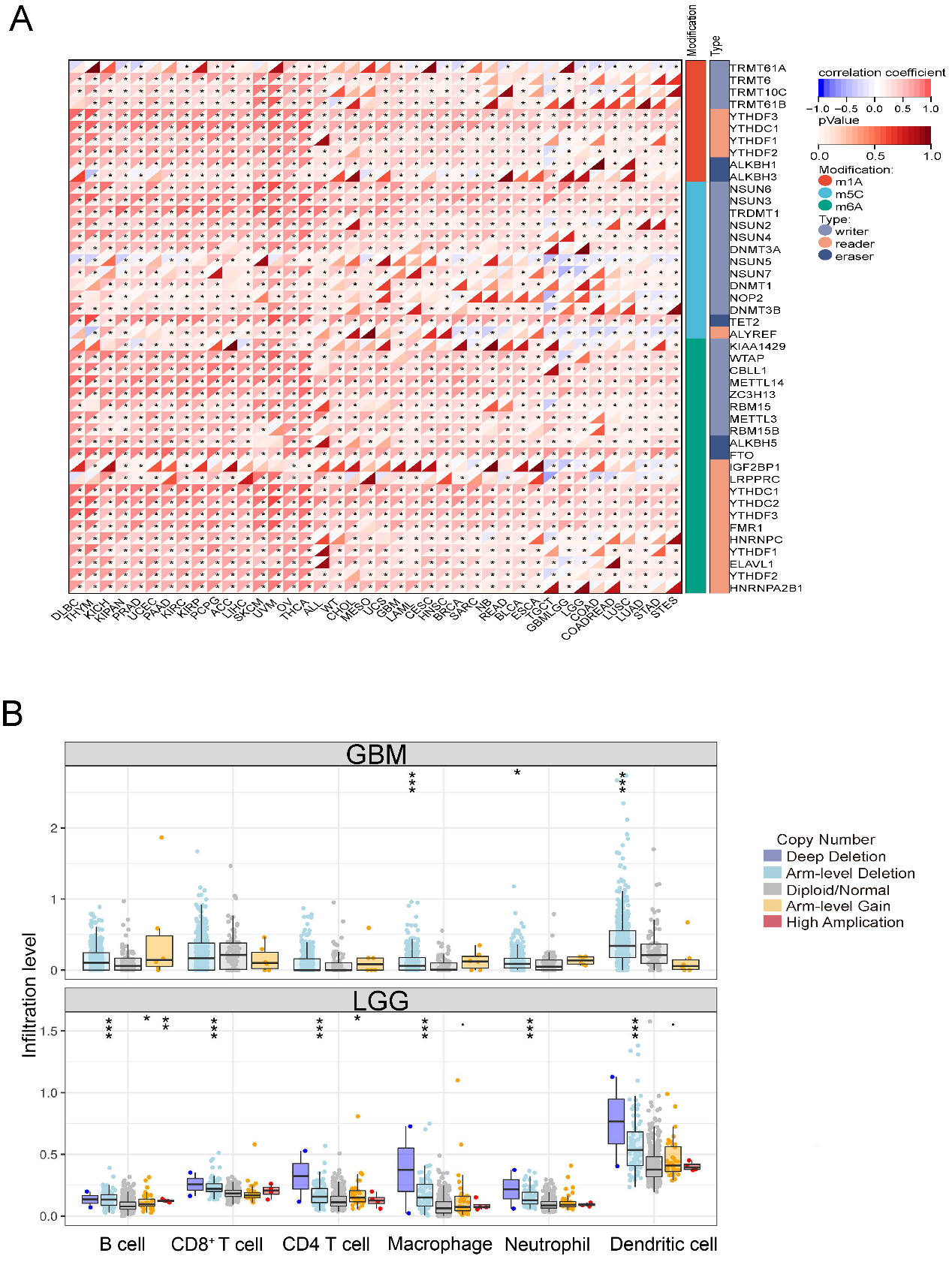


**Figure S3**. (A) The correlation between *ZNF25* expression and genes related to the functional proteins or enzymes involved in the writer, reader, and eraser of RNA modification. (B) The correlation between the CNA types of *ZNF25* and immune infiltration cells in GBM and LGG. (*p <0.05, **p< 0.01, ***p < 0.001)
